# Suppression of neuronal apoptosis and glial activation with modulation of Nrf2/HO-1 and NF-kB signaling by curcumin in streptozotocin-induced diabetic spinal cord central neuropathy

**DOI:** 10.3389/fnana.2023.1094301

**Published:** 2023-03-09

**Authors:** Hassan Reda Hassan Elsayed, Mohammed R. Rabei, Mohamed Mahmoud Abdelraheem Elshaer, Eman Mohamad El Nashar, Mansour Abdullah Alghamdi, Zainah Al-Qahtani, Ahmed Nabawy

**Affiliations:** ^1^Department of Anatomy and Embryology, Faculty of Medicine, Mansoura University, Mansoura, Egypt; ^2^Department of Anatomy, Faculty of Medicine, New Mansoura University, New Mansoura City, Egypt; ^3^Department of Medical Physiology, Faculty of Medicine, Mansoura University, Mansoura, Egypt; ^4^Department of Physiology, Faculty of Medicine, King Salman International University, South Sinai, Egypt; ^5^Department of Clinical Pharmacology, Faculty of Medicine, Ain Shams University, Cairo, Egypt; ^6^Department of Clinical Pharmacology, Faculty of Medicine, King Salman International University, South Sinai, Egypt; ^7^Department of Anatomy, College Medicine, King Khalid University, Abha, Saudi Arabia; ^8^Department of Histology and Cell Biology, Faculty of Medicine, Benha University, Benha, Egypt; ^9^Genomics and Personalized Medicine Unit, College of Medicine, King Khalid University, Abha, Saudi Arabia; ^10^Neurology Section, Internal Medicine Department, College of Medicine, King Khalid University, Abha, Saudi Arabia

**Keywords:** diabetic neuropathy, microglia, astrocytes, curcumin, Nrf2, HO-1, NF-kB

## Abstract

**Introduction:**

Diabetes is a global disease, commonly complicated by neuropathy. The spinal cord reacts to diabetes by neuronal apoptosis, microglial activation, and astrocytosis, with a disturbance in neuronal and glial Nuclear factor erythroid 2-related factor/Heme oxygenase-1 (Nrf2/HO-1) and Nuclear factor kappa-light-chain-enhancer of activated B cells (NF-kB) signaling. Curcumin, a bioactive natural substance, showed neuroprotective role in many diseases. However, its role in the treatment of the diabetic central neuropathy of spinal cord and the underlying mechanisms still need clarification. The present study tried to evaluate the role of curcumin in diabetes-induced central neuropathy of the spinal cord in rats.

**Methods:**

Twenty rats were divided into three groups; group 1: a negative control group; group 2: received streptozotocin (STZ) to induce type I diabetes, and group 3: received STZ + Curcumin (150 mg/kg/day) for eight weeks. The spinal cords were examined for histopathological changes, and immunohistochemical staining for Glia fibrillary acidic protein (GFAP); an astrocyte marker, Ionized calcium-binding adaptor molecule 1 (Iba1), a microglial marker, neuronal nuclear protein (NeuN); a neuronal marker, caspase-3; an apoptosis marker, Nrf2/HO-1, NF-kB, and oxidative stress markers were assessed.

**Results:**

Curcumin could improve spinal cord changes, suppress the expression of Iba1, GFAP, caspase-3, and NF-kB, and could increase the expression of NeuN and restore the Nrf2/HO-1 signaling.

**Discussion:**

Curcumin could suppress diabetic spinal cord central neuropathy, glial activation, and neuronal apoptosis with the regulation of Nrf2/HO-1 and NF-kB signaling.

## 1. Introduction

Today, there are 422 million diabetic patients. World Health Organization expects the number to become 700 million by 2045 (Saeedi et al., 2019). Peripheral, as well as, central neuropathies are common complications of diabetes. Treatment of diabetic neuropathy (DN) aims to provide control for blood glucose, manage pain, and suppress nerve damage. Currently, there is no efficient treatment for DN (Sloan et al., 2021).

Although many studies investigated the effect of diabetes on the peripheral nervous system (PNS) and trials to manage DPN, the diabetic-induced changes in the CNS, e.g., spinal cord, didn’t gain the same interest, and it must be studied to find new and effective treatments. Diabetes has been reported to cause microglial activation (Mandour et al., 2022; Wang et al., 2022), astrocytosis (Deng et al., 2017; Kiguchi et al., 2017), and neuronal apoptosis with up-regulation of BAX and Caspase-3 expressions (Mandour et al., 2022). Furthermore, diabetes causes up-regulation in the pro-inflammatory cytokine; Nuclear factor kappa light chain enhancer of activated B cells (NF-kB) (Mandour et al., 2022).

Nuclear factor erythroid 2-related factor (Nrf2)/Heme oxygenase-1 (HO-1) is a natural antioxidant cytoprotective system and a powerful modulator of longevity. This system can counteract oxidative stress, regulate apoptosis, modulate inflammation, and contribute to angiogenesis. When the cells face oxidative stress, Nrf2 translocates to nucleus, to regulate the genes transcription of anti-oxidant mediators (Lv et al., 2019). Diabetes causes a disturbance in Nrf2/HO-1 system (Pouso-Vazquez et al., 2022). Furthermore, the activation Nrf2 in neurons and/or neuroglia attenuated spinal cord ischemia-reperfusion injury through stimulating neuronal anti-oxidant and anti-apoptotic systems (Wang et al., 2017). Therefore, the oxidative stress and its subsequent neuronal apoptosis can be antagonized by activating Nrf2 pathway in damaged spinal cord.

A question was raised concerning whether modulating the spinal cord diabetic central neuropathy can affect directly the neuropathic pain. Zhang et al. could succeed in relieving diabetic neuropathic pain through reducing the spinal cord microglial activation (Zhang et al., 2018). In addition, Zhong et al. could alleviate diabetic neuropathic pain in rats by inhibiting spinal cord astrocyte activation (Zhong et al., 2018). Furthermore, Shayea et al. could control the diabetic neuropathic pain through the control of astrocyte activation and microglia-mediated inflammation (Shayea et al., 2020). Moreover, Basu et al. reviewed the successful role of modulation of spinal Nrf2/HO-1 system in controlling the peripheral neuropathic pain (Basu et al., 2022).

Curcumin, a primary bioactive substance in turmeric, has shown neuroprotective effects in a variety of diseases. Many studies reported the beneficial effect of Curcumin in diabetic peripheral neuropathy. It could attenuate neuropathic pain by inhibiting oxidative stress through suppression of NADPH oxidase, thus decreasing malondialdehyde (MDA) and increasing superoxide dismutase (SOD) activity (Zhao et al., 2014), through activation of the opioid system causing an antinociceptive effect (Banafshe et al., 2014), and through suppression of tumor necrosis factor (TNF) alpha expression (Daugherty et al., 2018). In addition, nano-curcumin supplementation could reduce depression and anxiety after diabetic neuropathy (Asadi et al., 2020). Furthermore, the action of curcumin in neuropathic pain may involve the pJNK pathway in the astrocytes and neurons of the dorsal root ganglia (DRG) (Park et al., 2021). Moreover, Curcumin could inhibit the apoptosis of Schwann cells (SCs) and could promote nerve growth factor (NGF) expression in sciatic nerves of diabetic peripheral neuropathy (DPN) rat model (Zhang et al., 2022). All the previous studies explored the role of Curcumin in diabetic peripheral neuropathy; however, few of these studies explored its role in diabetic central neuropathy.

Many studies reported the beneficial effect of curcumin on models of spinal cord injury (SCI) (Jin et al., 2021; Kahuripour et al., 2022). In addition, Curcumin could attenuate the hypoxia-induced white matter injury (Daverey and Agrawal, 2020). Furthermore, Curcumin could significantly reduce glial activation with down-regulation of spinal NF-kB and up-regulation of Nrf2 and HO-1 in Paclitaxel-treated rats with suppression of neuronal apoptosis (Yardim et al., 2021). However, the role of curcumin in the management of diabetes-induced spinal cord impairment still requires clarification.

The current study aimed for the first time to explored the role of curcumin against diabetes-induced central neuropathy in spinal cord, microglial activation, astrocytosis, neuronal apoptosis, and its role in the regulation of Nrf2/HO-1 and NF-kB signaling pathways.

## 2. Materials and methods

### 2.1. Ethical statement

The study was designed following the Animals in Research: Reporting *in Vivo* Experiments (ARRIVE) standards and meeting the standards of Mansoura University Animal Care and Use Committee (MU-ACUC), Egypt (MED.R.22.09.2).

### 2.2. Animals

Twenty adult male Sprague-Dawley (SD) rats weighing 150–200 grams, 7–8 weeks in age, were used. SD rats were used as they are considered efficient models for studying Type I diabetes-induced spinal cord injury (Inam et al., 2019; Shayea et al., 2020) and males were chosen because they have a greater degree of diabetic neuropathy, as compared to females (Fan et al., 2018). The animals were allowed to acclimate before the start of the experiment. They were kept in stainless steel cages, in firm day/night cycles, under appropriate temperature, and humidity, and in aseptic conditions, with a free source of food and water.

### 2.3. Research design

**FIGURE 1:**
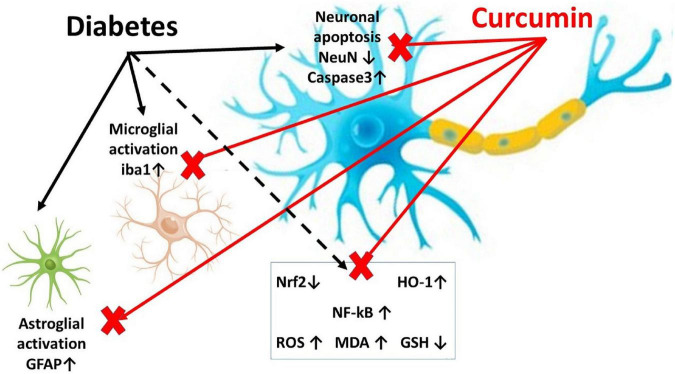
A graphical abstract demonstrating the research design.

The twenty animals were divided into three groups; Group 1 (vehicle control group; *n* = 6) received only 0.5% carboxymethylcellulose (CMC; the solvent of curcumin) by oral gavage once per day for 8 weeks. Group 2 (Diabetic; *n* = 8); after 12 h of fasting overnight, rats received an intra-peritoneal injection of freshly prepared streptozotocin (STZ; Sigma Aldrich, St. Louis, MO, USA), at a dose of 55 mg/kg of body weight to induce Type I Diabetes. STZ was dissolved in 0.1 M citrate buffer (pH = 4.5). Blood glucose was detected using an Accu check blood glucose meter (Roche Diagnostic, Germany) three days after STZ injection. The rats were confirmed diabetic when fasting blood glucose was >250 mg/dl, for two consecutive days. The rats received CMC once per day for 8 weeks after induction of diabetes. Group 3 (Diabetic + curcumin group; *n* = 6) received STZ, as mentioned above, and after induction of type I diabetes, the rats received Curcumin (Acros organics product of the US), at a dose of 150 mg/kg/day (Varatharajalu et al., 2016; Zheng et al., 2017; Ghelani et al., 2019) by oral gavage for 8 weeks. Curcumin suspensions in 0.5% carboxymethylcellulose were freshly prepared. Throughout the study, two rats from the diabetic group died. At the end of the study; 8 weeks after diabetes induction, the rats were subjected to sacrification through decapitation. Consequently, the spinal cords were removed, washed in saline, and dried. Figure 1 shows a graphical abstract for the study.

### 2.4. Detection of serum blood glucose, and oxidative stress markers (MDA and GSH)

Blood was taken from the hearts of the rats. Serum was separated. Serum glucose was detected using an Accu check blood glucose meter (Roche Diagnostic, Germany). MDA and GSH were measured following the technique of Elsayed et al. (2021a,b).

### 2.5. Assessment of histopathological changes and histopathological scoring

The spinal cords of the rats were excised and parts of cervical and lumbar segments were fixed in formaldehyde (10%) and then embedded in paraffin to evaluate the histopathological changes, then 7 μm thick sections were stained with hematoxylin and eosin (H&E) (Suvarna et al., 2018). Using the Olympus Light Microscope and SC100 camera, the dorsal horns of the cervical and lumbar segments were examined, as they are commonly affected by diabetic neuropathy and neuropathic pain. Semiquantitative histopathological scoring for the spinal cord changes was performed. Shrinkage of soma, neurons with piknotic nuclei, axon degeneration, inflammatory cell infiltrate, focal bleeding were evaluated and were graded as follows: 0, less than 5%; 1, 5–33%; 2, 34–66%; and 3, over 66%.

### 2.6. Immunohistochemical staining

Three μm thick sections of the spinal cord were processed for immunohistochemical staining using the immunoperoxidase method (Elhadidy et al., 2021; Elsayed et al., 2021a,^2022^). Concisely, the slides were deparaffinized and endogenous peroxidase was blocked. Hydrogen peroxide and 0.3% methanol were added to the spinal cord sections for 10 min at room temperature. To stimulate antigen retrieval, the sections were consequently subjected to heating at 95^°^C for 10 min in 10 mM citrate buffer and then left for 1 h to cool. The slides were kept with primary antibodies for NeuN; a neuronal marker, Iba1; a microglial marker, GFAP; an astrocyte marker, caspase-3; an apoptosis marker, Nrf2/HO-1, and NF-kB inflammatory and oxidative stress markers, overnight at 4^°^C. Table 1 presents the details of the antibodies and their dilutions. Consequently, the slides were kept for 30 min with a mouse-rabbit polydetector (BSB 0268, Bioscience). For the reagent (no-primary antibody) control, Phosphate-buffered saline (PBS), was added as a substitute for the primary antibody. Lastly, the slides were washed, then dehydrated, and investigated with a light microscope (Ramos-Vara and Miller, 2014). Dark brown areas on a blue background, demonstrate positive staining. Antigen localization was mainly nuclear for NeuN, mainly cytoplasmic for GFAP, cytoplasmic, and nuclear for caspase-3, Iba1, Nrf2, HO-1, and NF-kB.

**TABLE 1:** Primary antibodies applied for immunohistochemistry.

Name	Cat. number	Source and clonality	Dilution
NeuN	ABclonal A19086	Rabbit monoclonal	1/100
Iba1	ABclonal A19776	Rabbit monoclonal	1/100
GFAP	Servicebio GB11096	Rabbit polyclonal	1/1000
Caspase-3	Servicebio GB11532	Rabbit polyclonal	1/500
Nrf2	ABclonal A11159	Rabbit polyclonal	1/100
HO-1	Santa Cruz sc-390991	Mouse monoclonal	1/200
NF-kB	ABclonal A19653	Rabbit monoclonal	1/100

### 2.7. Morphometric analysis

This was performed utilizing the 1.52a version of ImageJ software (Schneider et al., 2012) and Fiji ImageJ software (Schindelin et al., 2012). The number of NeuN, Iba1, GFAP, Caspase-3, Nrf2, HO-1, and NF-kB immunopositive cells/high-power field (x400) was counted in spinal cord sections from rats of all groups.

### 2.8. Statistical analysis of immunohistochemical results

Data were analyzed utilizing IBM-SPSS software. After normality testing, normal quantitative data from the three study groups were compared using one-way ANOVA and *Post-hoc* Tukey test. The data that are not normally distributed were presented as median and interquartile range and a Kruskal–Wallis H test was used to compare them. The results were considered significant if the *p*-values < 0.050.

## 3. Results

### 3.1. Effect of curcumin treatment on serum blood glucose, MDA, and GSH

**FIGURE 2:**
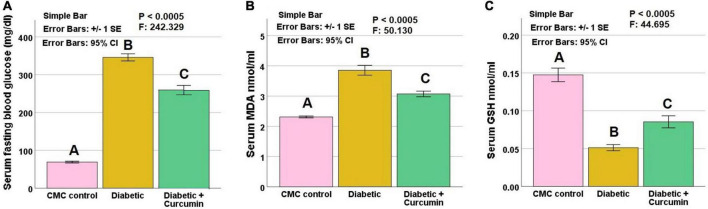
**(A)** Serum glucose, **(B)** Serum MDA, and **(C)** Serum GSH, in the studied groups. Histograms show means ± standard errors (SE). Data are mentioned as mean ± SE, different letters = significant difference. *P* is significant if < 0.05. MDA, malondialdehyde; GSH, reduced glutathione.

Serum glucose, MDA, and GSH revealed significant differences between the studied groups (*p*: < 0.0005). The diabetic group revealed significantly higher serum glucose, and MDA levels, as well as, a significantly lower level of GSH compared to the control group. These findings were reversed by curcumin administration compared to the diabetic group. On the other hand, serum glucose, MDA, and GSH levels, still revealed a significant difference from the control group (Figure 2).

### 3.2. Effect of curcumin administration on diabetes-induced histopathological alteration and histopathological score in the dorsal horn of the cervical and lumbar segments of the spinal cord

**FIGURE 3:**
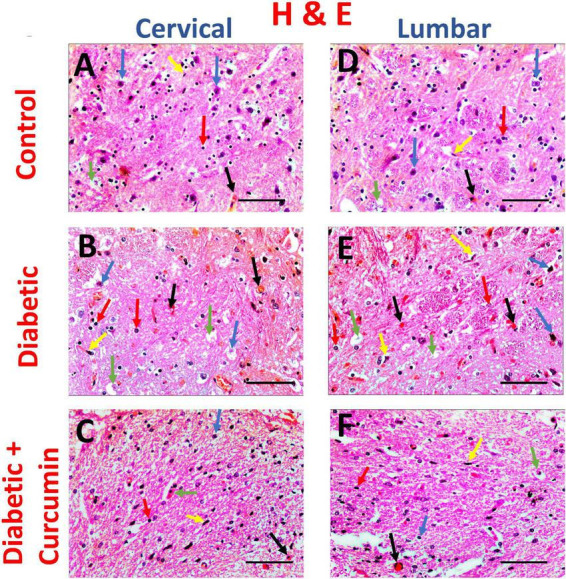
Impact of curcumin on histopathological changes in the dorsal horn of cervical and lumbar segments in diabetic rats by H & E (×400. Scale bar = 50 μm. The control group **(A,D)** revealed medium-sized basophilic somas of the sensory neurons (blue arrows) in acidophilic neutropil, myelinated axons with their myelin sheaths (green arrows), together with few glial cells; microglia (yellow arrows) and astrocytes (red arrows). Blood capillaries appear intervening (black arrows). The Diabetic group **(B,E)** revealed shrinkage of neuronal somas with surrounding haloes and pyknotic nuclei (blue arrows), degenerated axons (green arrows), multiple microglia (yellow arrows), and astrocytes (red arrows). Focal areas of hemorrhage (black arrows) are noticed. The Curcumin-treated group **(C,F)**, revealed relatively normal neuronal somas, with few shrunken somas (blue arrows) in acidophilic neuropil. Relatively normal myelinated nerve axons, with few degenerated axons (green arrows), together with few glial cell nuclei; microglia (yellow arrows) and astrocytes (red arrows). Few areas of congestion (black arrows) are noticed.

The negative control group (Figures 3A, D) revealed medium-sized basophilic neuronal somas, and myelinated axons with their myelin sheaths, together with few glial cells; microglia, and astrocytes. Blood capillaries appear intervening. The diabetic group (Figures 3B, E) revealed shrunken neuronal somas with surrounding haloes and pyknotic nuclei, degenerated nerve axons, and myelin sheaths, together with multiple microglia, and astrocytes. Focal areas of bleeding are noticed. The Curcumin-treated group (Figures 3C, F), revealed relatively normal neuronal somas, with few shrunken neuronal somas. Relatively normal myelinated axons, and myelin sheaths with few degenerate axons, together with few glial cell nuclei; microglia (yellow arrows) and astrocytes. Few areas of congestion are noticed. The histopathological score showed a statistically significant higher scores in the diabetic group (median value = 9) as compared to the control groups (median value = 1), with a significant reduction in the curcumin-treated group (median values = 3.5) when compared to the diabetic group. Table 2 shows the results of the histopathological score.

**TABLE 2:** Effect of curcumin on the histopathological scoring for spinal cord changes, induced by diabetes.

	Control (*n* = 6)	Diabetic (*n* = 6)	Diabetic + Curcumin (*n* = 6)	H value	*p*-value
Shrinkage of soma	0 (0–0) A	2 (1.25–2) B	1 (0–1) A	41.466	<0.0005
Neurons with pyknotic nuclei	0 (0–0) A	2 (1–2) B	1 (0–1) C	37.135	<0.0005
Axon degeneration	0 (0–0.75) A	2 (1.25–2) B	1 (0–1) A	37.098	<0.0005
Inflammatory cell infiltrate	0 (0–1) A	2 (1.25–2) B	1 (0–1) A	35.871	<0.0005
Focal bleeding	0 (0–0.75) A	2 (1.25–2) B	1 (0–1) A	38.640	<0.0005
Score	1 (1–1.75) A	9 (8–9) B	3.5 (3–4.75) C	52.100	<0.0005

Results are presented as median and interquartile range. The *p*-value was determined by the Kruskal–Wallis H test; different letters = statistically significant difference. Significant *p*-values (≤0.05).

### 3.3. Immunohistochemical results

**FIGURE 4:**
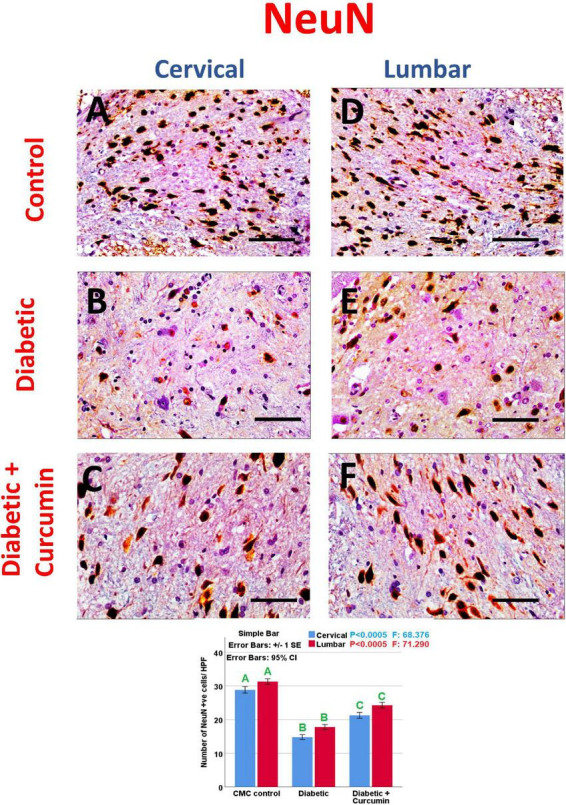
**(A–C)** Cervical and **(D–F)** lumbar. Impact of curcumin on the immunohistochemical expression of NeuN in the dorsal horns of spinal cords of diabetic rats (×400). Scale bar = 50 μm. Histogram shows the impact of Curcumin on the number of NeuN, +ve cells/HPF, in the dorsal horns of spinal cords of diabetic rats. Results are mentioned as mean ± standard error. The results were compared using one-way ANOVA and *Post-hoc* Tukey test. The results were considered significant if the *p*-values < 0.050. Different letters mean significant differences. HPF, high power field.

**FIGURE 5:**
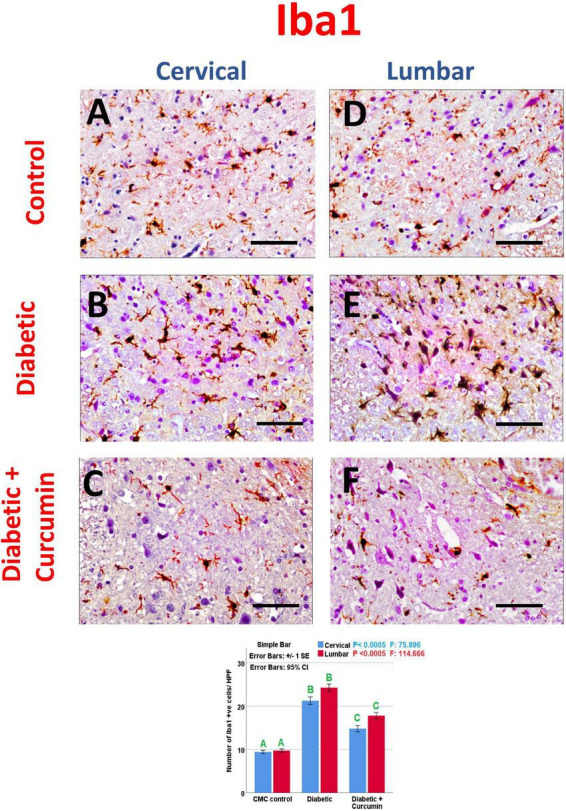
**(A–C)** cervical and **(D–F)** lumbar. Impact of curcumin on the immunohistochemical expression of Iba1 in the dorsal horns of spinal cords of diabetic rats (×400). Scale bar = 50 μm. Histogram shows the impact of Curcumin on the number of Iba1, +ve cells/HPF, in the dorsal horns of spinal cords of diabetic rats. Results are mentioned as mean ± standard error. The results were compared using one-way ANOVA and *Post-hoc* Tukey test. The results were considered significant if the *p*-values < 0.050. Different letters mean significant differences. HPF, high power field.

**FIGURE 6:**
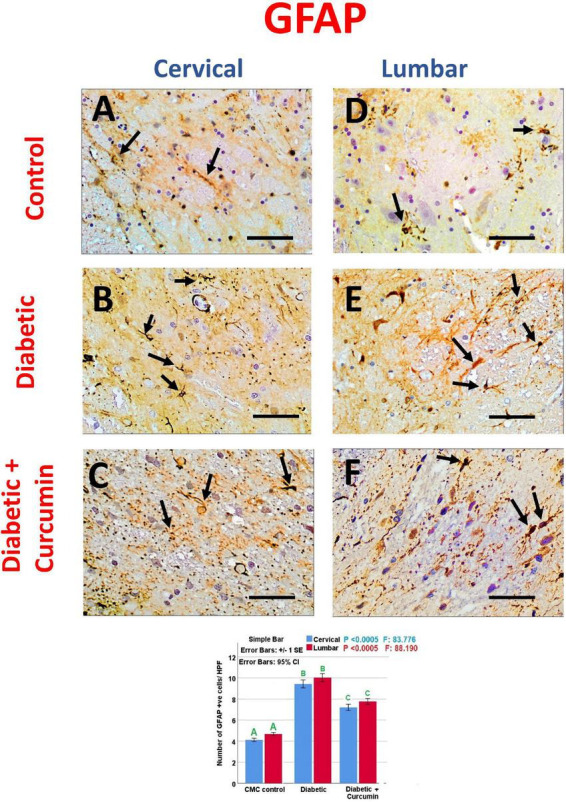
**(A–C)** cervical and **(D–F)** lumbar. Impact of curcumin on the immunohistochemical expression of GFAP in the dorsal horns of spinal cords of diabetic rats (×400). Scale bar = 50 μm. Histogram shows the impact of Curcumin on the number of GFAP, +ve cells/HPF, in the dorsal horns of spinal cords of diabetic rats. Results are mentioned as mean ± standard error. The results were compared using one-way ANOVA and *Post-hoc* Tukey test. The results were considered significant if the *p*-values < 0.050. Different letters mean significant differences. HPF, high power field.

**FIGURE 7:**
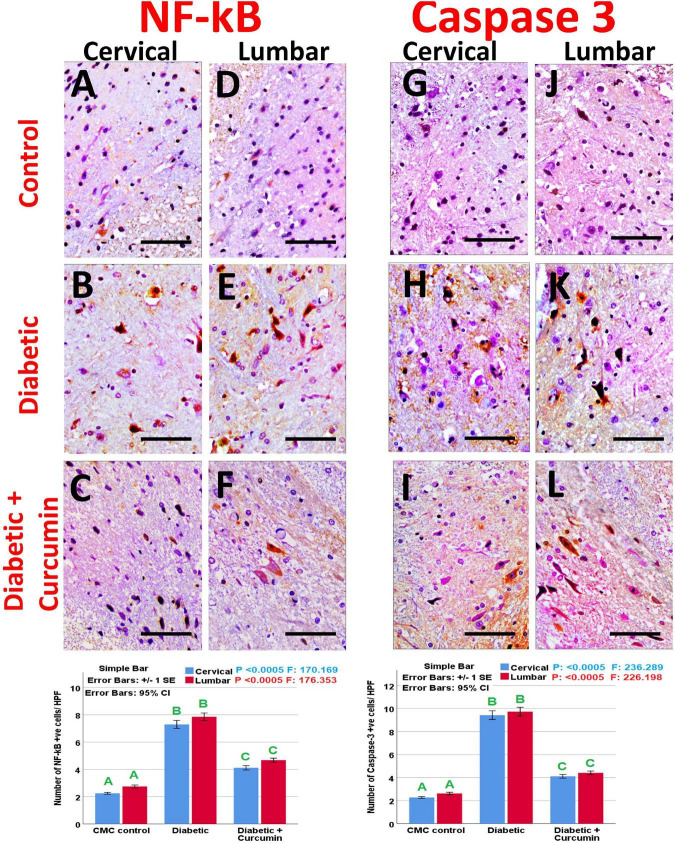
**(A–C)** NF-kB expression in cervical, **(D–F)** NF-kB expression in lumbar, **(G–I)** caspase 3 expression in cervical, and **(J–L)** caspase 3 expression in lumbar. Impact of curcumin on the immunohistochemical expression of NF-kB and caspase-3 in the dorsal horns of spinal cords of diabetic rats (×400). Scale bar = 50 μm. Histograms shows the impact of Curcumin on the number of NF-kB and caspase-3, +ve cells/HPF, in the dorsal horns of spinal cords of diabetic rats. Results are mentioned as mean ± standard error. The results were compared using one-way ANOVA and *Post-hoc* Tukey test. The results were considered significant if the *p*-values < 0.050. Different letters mean significant differences. HPF, high power field.

**FIGURE 8:**
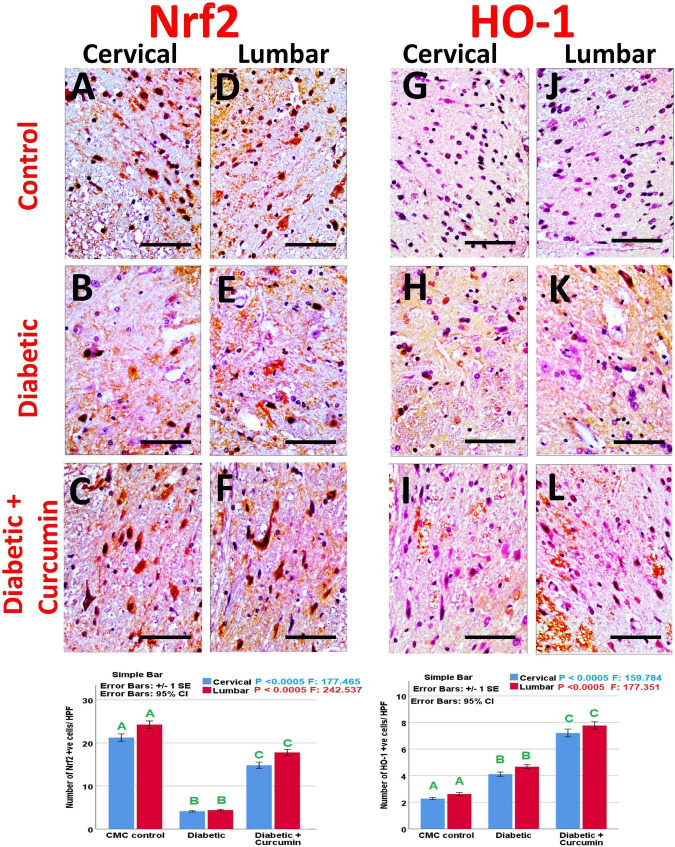
**(A–C)** Nrf2 expression in cervical, **(D–F)** Nrf2 expression in lumbar, **(G–I)** HO-1 expression in cervical, and **(J–L)** HO-1 expression in lumbar. Impact of curcumin on the immunohistochemical expression of Nrf2 and HO-1 in the dorsal horns of spinal cords of diabetic rats (×400). Scale bar = 50 μm. Histograms shows the impact of Curcumin on the number of Nrf2 and HO-1 + ve cells/HPF, in the dorsal horns of spinal cords of diabetic rats. Results are mentioned as mean ± standard error. The results were compared using one-way ANOVA and *Post-hoc* Tukey test. The results were considered significant if the *p*-values < 0.050. Different letters mean significant differences. HPF, high power field.

Immunohistochemical detection of NeuN, Iba1, GFAP, Caspase-3, Nrf2, HO-1, and NF-kB positive cells, of the negative control group showed moderate immunoreactivity for NeuN, Iba1, GFAP, mild expression for Nrf2, and HO-1 with weak expression for Caspase3 and NF-kB. Noticeably, the dorsal horns of the spinal cords of the diabetic group revealed a strong expression for Iba1, GFAP, NF-kB, and Caspase3, with moderate expression for HO-1, and a mild expression for NeuN and Nrf2. In contrast, the diabetic + Curcumin group revealed reversed immunoreactivity with moderate immunoreactivity for NeuN, Iba1, GFAP, and Nrf2, with a weak expression for caspase3, and NF-kB. However, there was a strong expression for HO-1 (Figures 4–8).

### 3.4. Results of morphometric analysis of immunohistochemical results

The number of NeuN, Iba1, GFAP, Caspase-3, Nrf2, HO-1, and NF-kB immunopositive cells, revealed a significant difference (*p* < 0.0005) between the studied groups. Tukey *post-hoc* tests showed a significant increase in the number of Iba1, GFAP, NF-kB, caspase3, and HO-1 positive cells with a significant reduction in the number of NeuN and Nrf2 positive cells in the diabetic group as compared to the negative control group. Furthermore, a significant reduction in the number of Iba1, GFAP, Caspase3, and NF-kB positive cells, as well as a significant increase in the number of NeuN, Nrf2, and HO-1 positive cells, were observed in the diabetic + Curcumin group, when compared to the diabetic group. However, the results of the curcumin-treated group revealed a significant difference compared with the control group (Figures 4–8).

## 4. Discussion

Peripheral, as well as, central neuropathies are common complications of diabetes. Treatment of diabetic neuropathy (DN) aims to provide control for blood glucose, manage pain, and suppress nerve damage. Currently, there is no efficient treatment for DN (Sloan et al., 2021). Curcumin (Turmeric), a primary bioactive substance, derived from Curcuma Longa, has shown neuroprotective effects in many diseases. Many studies reported the beneficial effect of Curcumin on diabetic peripheral neuropathy (Zhang et al., 2022) and spinal cord traumatic injury models (Kahuripour et al., 2022). However, the role of curcumin in the management of diabetes-induced spinal cord impairment requires clarification. The current study explored the role of curcumin against diabetes-induced spinal cord microglial activation, astrocytosis, neuronal apoptosis, and its role in the regulation of the Nrf2/HO-1 and NF-kB signaling pathways.

In the present study, STZ could induce type I diabetes, followed by the development of central neuropathy in spinal cord. Diabetes induced oxidative stress with decreased GSH and increased MDA, consistent with Mandour et al. (2022) who reported similar findings. The current study also found that diabetes-induced spinal cord microglial activation, as seen by up-regulation of Iba1 expression, is consistent with the findings of Mandour et al. (2022) and Wang et al. (2022), as microglia are the main provider of inflammatory cytokines; TNF-α, IL-6, and IL-1β, in response to neuronal degeneration. On the other hand, astrocytes; the star-shaped neuroglia in CNS, perform a nutritional function, preserve the ion balance, regulate the blood flow to the brain and perform a trial to repair, otherwise, scarring of CNS after injury. The present study found that diabetes could induce spinal cord astrocytosis, as manifested by increased expression of GFAP, similar to the finding of Dauch et al. (2012), Benitez et al. (2015), Deng et al. (2017), and Kiguchi et al. (2017), however, Wodarski et al. (2009) and Shayea et al. (2020) found that STZ rats had a reduced number of astrocytes. Interestingly, Tsuda et al. (2008) and Zhang et al. (2018) found an insignificant change in the number of astrocytes in the diabetic spinal cord. The controversy surrounding these findings may be due to the difference in the model, the dose of STZ, and the duration of diabetes.

NeuN is a neuronal marker with a nuclear expression. In the current study, the number of NeuN positive cells was found to decrease in the spinal cords of STZ rats suggesting a reduced neuronal number. This decrease may be at least in part due to neuronal apoptosis as demonstrated by increased caspase3 expression in the diabetic spinal cord group, similar to the results of Inam et al. (2019), Niknia et al. (2019), and Mandour et al. (2022).

The mechanisms underlying neuronal apoptosis may be the disruption of the Nrf2/HO-1 and NF-kB pathways. It is a cytoprotective system and a powerful modulator of longevity. This pathway can counteract oxidative stress, regulate apoptosis, modulate inflammation, and contribute to angiogenesis. The present study reports down-regulation of Nrf2 with up-regulation in the expression of HO-1, consistent with the findings of Pouso-Vazquez et al. (2022) with the induction of NF-kB pro-inflammatory pathway similar to the results of Mandour et al. (2022). However, Castany et al. (2016) reported no change in HO-1 in diabetes. NF-kB has been reported to co-localize with GFAP, suggesting the role of astrocytes in the regulation of NF-kB activity (Lee et al., 2011). On the other hand, Nrf2 was found to co-localize with Iba1, GFAP, and NeuN, confirming its role in microglia, astrocytes, and neurons, respectively, after spinal cord trauma (Wang et al., 2017).

Curcumin, a primary bioactive substance in turmeric, has shown neuroprotective effects in a variety of diseases. In the present study, Curcumin was found to protect the spinal cord against diabetes-induced injury. Many studies reported the beneficial effect of Curcumin on diabetic peripheral neuropathy through antioxidant activity (Zhao et al., 2014), activation of the opioid system (Banafshe et al., 2014), suppression of TNF alpha expression (Daugherty et al., 2018), reduction of depression and anxiety (Asadi et al., 2020), modulation of the activity of DRG astrocytes and neurons (Park et al., 2021), inhibition of Schwann cell apoptosis and promoting nerve growth factor (NGF) (Zhang et al., 2022). On the other hand, many studies reported the beneficial role of curcumin in the treatment of injury, induced by spinal cord trauma models (SCI) (Kahuripour et al., 2022).

In the present study, curcumin could suppress diabetes-induced microglial activation, consistent with the results of Wang et al. (2014) who found that curcumin could promote spinal cord repair by suppressing microglia, thus inhibiting glial scar formation and the inflammatory response following a traumatic injury to the spinal cord, and also consistent with the results of Sheikholeslami et al. (2019), who described the beneficial role of curcumin in antagonizing morphine dependence, by inhibiting the microglial activation and decreasing the inflammatory mediators. In the present study, curcumin could attenuate the astrocytosis induced by diabetes, similar to the findings of Daverey and Agrawal (Daverey and Agrawal, 2020) who reported the role of Curcumin in the down-regulation of the hypoxia-induced astrocytosis as demonstrated by the expression of GFAP, in white matter hypoxic injury (WMI), and similar to the results of Yardim et al. (2021), as they described the role of curcumin in reducing astrocytosis in the spinal cord of Paclitaxel-treated rats.

Furthermore, the present study found that curcumin could rescue the spinal cord neurons from diabetes-induced injury as demonstrated through the restoration of the number of NeuN positive cells, consistent with the results of Lin et al. (2011), who described the role of curcumin in the restoration of NeuN positive neurons after neuronal loss after traumatic spinal cord injury. The mechanisms underlying this role for curcumin may be its antiapoptotic activity that was demonstrated in this study through decreased caspase-3 activity, similar to the results of Hao et al. (2017) and Xi et al. (2019) in models of traumatic spinal cord injury, Daverey and Agrawal (2020) in the model of spinal cord white matter hypoxic injury (WMI), and Yardim et al. (2021) in the spinal cord of Paclitaxel-treated rats. Moreover, Li et al. (2021) reported that Curcumin could promote functional recovery and reduce the number of apoptotic neurons after spinal cord trauma by modulating autophagy.

The anti-apoptotic effect of curcumin, as reported here, may be due to its role in the restoration of the Nrf2/HO-1 and NF-kB pathways. The current study found that curcumin could up-regulate Nrf2 and HO-1 expressions and down-regulate NF-kB expression in the diabetic spinal cord and could increase GSH and decrease MDA levels. Jin et al. (2021) reported a similar finding that Curcumin could rescue the spinal cord after traumatic injury by activating Nrf2/HO-1 and scavenging free radicals. Furthermore, Yardim et al. (2021) found that Curcumin could significantly up-regulate spinal Nrf2 and HO-1 expressions and reduce the expression of NF-kB, TNF-alpha, IL-6, and iNOS in Paclitaxel-treated rats.

The current study found that Curcumin could exert an antihyperglycemic effect on Diabetic rats, another mechanism that helps the neuroprotective role of Curcumin, however, it did not normalize serum glucose, similar to previous reports by Daugherty et al. (2018) and Zhang et al. (2022).

## 5. Conclusion

Curcumin could improve spinal cord changes- induced by diabetes. It could suppress microglial activation, astrocytosis, and neuronal apoptosis with the restoration of the normal activity of Nrf2/HO-1 and NF-kB. Curcumin is a promising adjuvant therapy to suppress diabetes-induced spinal cord microglial activation, astrocytosis, and neuronal apoptosis through regulation of the Nrf2/HO-1 and NF-kB signaling pathways.

### 5.1. Study limitations

Sprague Dawley rats were used as they are considered efficient models for studying Type I diabetes-induced spinal cord injury (Inam et al., 2019; Shayea et al., 2020) and males were chosen because they have a greater degree of diabetic neuropathy, as compared to females (Fan et al., 2018). So the controversy surrounding the effect of STZ and/or Curcumin on the spinal cord neurons, glia, Nrf2/HO-1, and NF-kB signaling may be due to the difference in sex or species as well as the difference in STZ and curcumin dosage and/or duration, and even the type of diabetes. Furthermore, it may be due to the different segments of spinal cord or horns examined. To better validate the results, further studies should try several doses of STZ and Curcumin, various animals and species, different sex, different regimens, different segments and horns of spinal cord and even more diabetic models.

### 5.2. Clinical application

The present study recommends the use of Curcumin as an adjuvant to suppress diabetic spinal cord central neuropathy, glial activation, and neuronal apoptosis with the regulation of Nrf2/HO-1 and NF-kB signaling.

## Data availability statement

The raw data supporting the conclusions of this article will be made available by the authors, without undue reservation.

## Ethics statement

The study was designed following the Animals in Research: Reporting *In Vivo* Experiments (ARRIVE) standards and meeting the standards of Mansoura University Animal Care and Use Committee (MU-ACUC), Egypt (MED.R.22.09.2).

## Author contributions

HE and AN: conceptualization, methodology, validation, investigation, data curation, and writing—original draft preparation. MR and ME: software. HE, AN, MR, and ME: formal analysis and visualization. MR, ME, EE, MA, and ZA-Q: resources. HE, MR, ME, EE, MA, and ZA-Q: writing—review and editing. EE, MA, and ZA-Q: supervision and project administration. All authors have read and agreed to the published version of the manuscript.
